# Three Waves of Data Use Among Health Workers: The Experience of the Better Immunization Data Initiative in Tanzania and Zambia

**DOI:** 10.9745/GHSP-D-19-00024

**Published:** 2019-09-23

**Authors:** Laurie Werner, Dawn Seymour, Chilunga Puta, Skye Gilbert

**Affiliations:** a PATH, Seattle, WA, USA.; b PATH, Lusaka, Zambia. Now with Digital Impact Alliance, Washington, DC, USA.; c PATH, Lusaka, Zambia.

## Abstract

The governments of Tanzania and Zambia identified key data-related challenges affecting immunization service delivery including identifying children due for vaccines, time-consuming data entry processes, and inadequate resources. To address these challenges, since 2014, the countries have partnered with PATH's Better Immunization Data Initiative to design and deploy a suite of data quality and use interventions. Two key aspects of the interventions were an electronic immunization registry and tools and practices to strengthen a culture of data use. As both countries deployed the interventions, 3 distinct changes in data use emerged organically. This article provides a detailed summary of these 3 phases or waves, based mostly on qualitative data or observation: (1) strengthening data collection using new data collection tools and processes and increasing efficiency of health workers; (2) improving data quality regarding accuracy and completeness; and (3) increasing use of data to take action to strengthen their work and for programmatic decision making. These waves clearly demonstrated the growing ability of health workers to move from data collectors to data analyzers who began to focus on the data quality and then the value of using the data in their day-to-day activities.

## Abstract (precis)

Data quality and use rollout in Tanzania's and Zambia's immunization programs progressed along 3 phases—from strengthening data collection, to improving data quality, to increasing data use for programmatic decision making cultivating a culture of data use.

## INTRODUCTION

Over the past 2 decades, although global immunization coverage rates have increased with more children being protected by these lifesaving interventions, disparities at the subnational level are becoming more apparent.^1^^,^^2^ Global partners, such as the World Health Organization and United Nations Children's Fund, are going to great lengths to understand these inequities at the global level. However, poor data availability and quality sometimes limits both global and national-level programs from understanding these inequities and how to address them. This article describes work undertaken through the Better Immunization Data (BID) Initiative, led by PATH, to work in partnership with the governments of Tanzania and Zambia to improve data collection, quality, and use, with the ultimate goal of enhancing immunization and overall health service delivery. This work has been grounded in the belief that better data plus better decisions will lead to better health outcomes.

Both Tanzania and Zambia have seen their national coverage rates increase, but continue to face challenges with regions having low coverage.^3^ In 2017, Tanzania and Zambia reported coverage rates for the third dose of the diphtheria, tetanus, and pertussis (DTP3) vaccine of 97% and 94%, respectively, yet the subnational data show areas with coverage as low as 60%–70%. When the BID Initiative work began in 2014, Tanzania and Zambia government stakeholders identified several challenges with data use in their health systems that contributed to these inequities^4^:

- **Unnecessarily vulnerable children.** In a paper-based system, health workers had difficulty identifying children due for vaccines as well as children who had missed vaccines (defaulters). It was also difficult to ensure that the necessary vaccine supplies would be available in adequate quantities at the appropriate time.
- **Time away from patients.** Recording the same or similar data sets across multiple forms during immunization services (as well as producing monthly reports for the district level) consumes time, especially when 1 or 2 health workers vaccinate hundreds of children.
- **Wasted and/or inadequate resources.** Without high-quality and accessible data, health workers struggled to accurately plan outreach services and the distribution of stock and supplies, leading to vaccine stock-outs or, alternatively, vaccine wastage.

**FIGURE 1:**
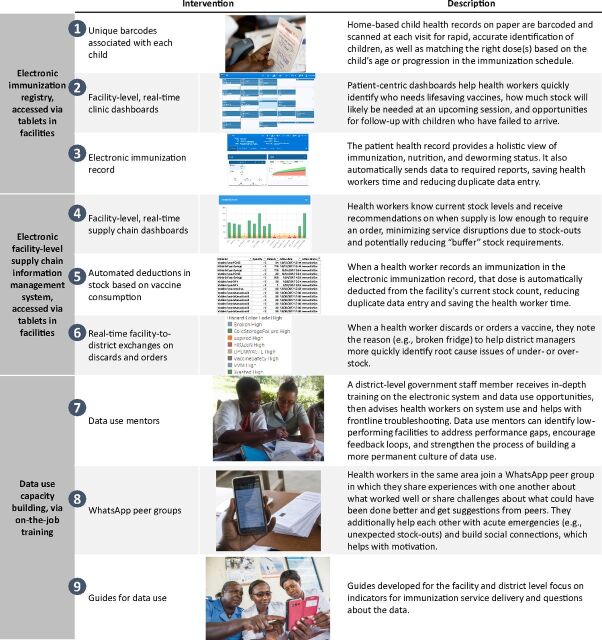
Data Quality and Use Interventions Developed Under the Better Immunization Data Initiative Work

The BID Initiative, building on periodic literature reviews, a dynamically updated theory of change, and frequent consultations with user advisory groups in Tanzania and Zambia, developed a suite of data quality and use interventions to address these challenges and to begin to build a data use culture to “support and encourage the use of evidence, including facts, figures, and statistics, to inform their decision making” (Figure 1).^5^^–^^12^ The user advisory groups included immunization health workers from all levels of the health system. The suite included other interventions targeted at building a data use culture, such as using data to target supportive supervision, data use campaigns and peer-support networks to support data use, and a change management strategy used throughout implementation (described later in this article). The interventions were tested in facilities and districts and developed iteratively before a final suite of complementary interventions were compiled for implementation and scale-up in each country. The interventions were intended to give the power of data to health workers, so they can do their jobs more effectively and efficiently.

The interventions were intended to give the power of data to health workers, so they can do their jobs more effectively and efficiently.

As of March 2018, the Tanzania Electronic Immunization Registry and data use interventions are in use at all facilities that provide immunizations in 4 regions (Arusha, Dodoma, Kilimanjaro, and Tanga). The Government of Tanzania plans to deploy to another 10 regions in 2018 and scale nationally in 2019. In Zambia, the government deployed the Zambia Electronic Immunization Registry and data use interventions to all facilities providing immunizations in Southern Province, with the government planning scale-up and sustainability efforts. PATH continues to partner with both governments as they continue to scale the interventions.

This article focuses specifically on the data use culture and the 3 distinct data use “waves” (or phases) that emerged organically and were observed qualitatively during implementation as health workers became more empowered to use data. These waves bear some similarity to those observed in the electronic Vaccine Intelligence Network pilot in India.^11^^-^^13^ It is important to understand how individuals and cultures go through phases as they build and strengthen data use and to consider these phases when planning any implementation effort focused on building a culture of data use, even knowing that each individual will move through them at their own specific pace. In particular, the phases can help align expectations on what results to expect when and where an implementation is in its critical path toward achieving stable, routine data use.

Each individual will move through the phases at their own pace as they build and strengthen data use.

## THREE WAVES OF PROGRESS TOWARD DATA USE

**FIGURE 2:**
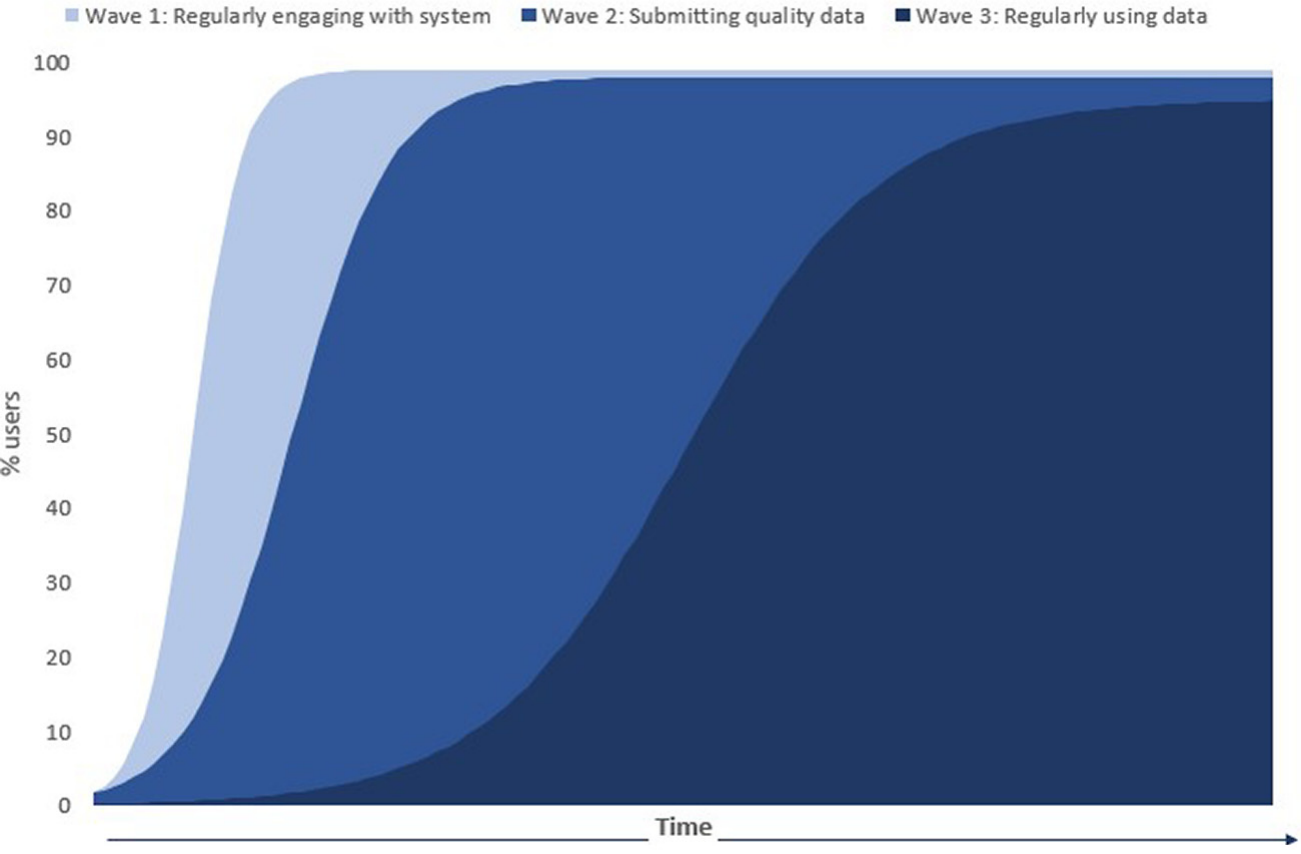
Illustrative Depiction of Health Workers Progressing Through 3 Waves of Data Use

The implementations in both countries consisted of 4 visits to the facilities to train health workers on the interventions. In Tanzania, the visits in the initial region began in 2015, and in Zambia, they began in 2017. Over several months, as the implementation progressed beyond its initial stages and as expected, the BID Initiative team observed health workers progress through 3 data use waves (Figure 2):

1. Strengthening data collection
2. Improving data quality
3. Increasing use of data for programmatic decision making

Depending upon the capacity of the health workers, it took on average approximately 2–3 months to proceed through the all the waves, with the first wave taking 2-3 weeks, the second wave taking another 2–3 weeks, and the third wave occurring after the health workers were familiar enough with the tool and the ways to use it to improve data quality.

### First Wave: Strengthening Data Collection

In the initial wave, health workers focused on receiving and learning how to use the new data collection tools and processes. The BID Initiative provided tablets for health workers to enter data into the electronic immunization registry (EIR). Health workers mostly expressed excitement to use the new tools (with some initial reluctance from those with less experience using digital tools like mobile phones), especially tablets or any digital devices. The tablets represented a tangible change in workflow efficiency, data accessibility, and a transition from legacy paper systems to digital health solutions.

**Figure:**
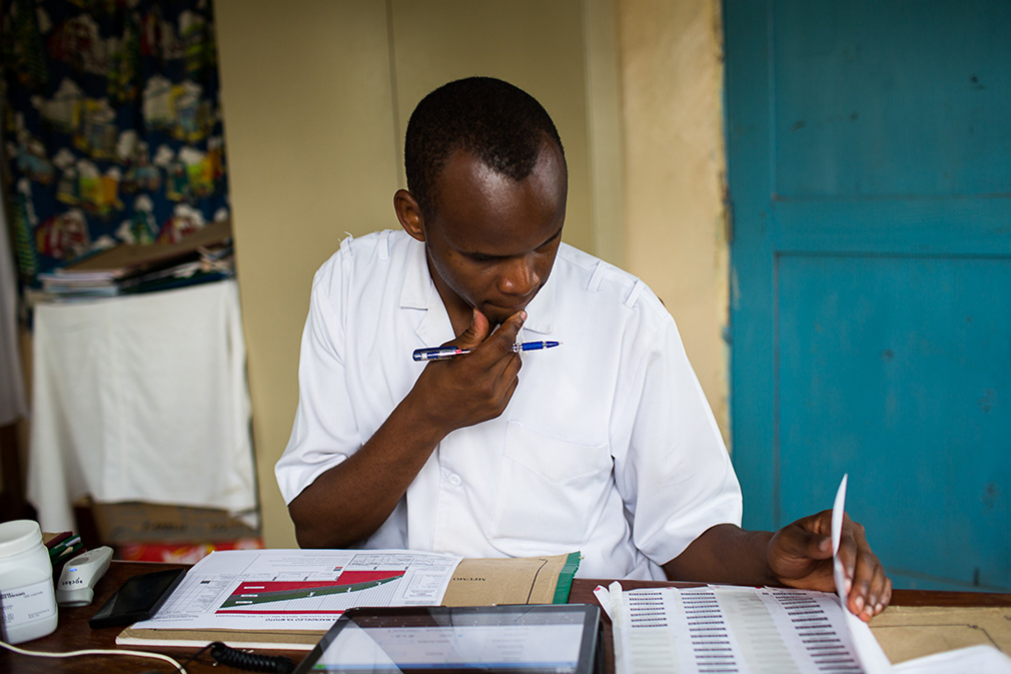
Paul Urioh, a health worker at Mareu Health Center in Tanzania, reviews patient data in Tanzania's electronic immunization registry. © 2015 Trevor Snapp/PATH

During this phase, the implementation team adapted the training based on the health workers' proficiency with digital tools. Health workers who owned smartphones easily adopted the new digital tools, but for health workers who did not own smartphones or who preferred the legacy systems, the training was tailored to familiarize them with the device as well as address resistance to change. Health workers reported that it took 2–3 weeks to feel proficient with the new tools.

In both countries, the EIR systems were developed on open-source software platforms using a user-centered design approach: Open Immunize (Tanzania)^14^ and Open Smart Register Platform (Zambia).^15^ They were designed to be used on Android tablets with offline functionality, ability to work in low-resource settings, and ability to register and track children through their vaccine schedule. Each child was given a unique identification number and barcode (2D in Tanzania, QR in Zambia) to facilitate searching for and managing their record. The key data collected by the systems included demographic information for the child and their caregiver (including their contact information to allow for follow-up if a child missed a vaccine dose), information on each visit or encounter (such as which antigens were given to the child), information on the facility and provider, data on the stock used for immunization services (vaccines and associated supplies), as well as other key indicators normally tracked on the child health register such as Vitamin A, use of bed nets, and the child's weight. Each EIR was developed with interoperability in mind to ensure the ability to share key data with the national health management information system and other systems present in each country, and effectively both systems can share data with the national health management information system and other information systems. The government information, communication, and technology and monitoring and evaluation departments were deeply involved throughout the process to ensure alignment with national policies and strategies as the systems were developed. These systems were essential to address the identified challenges and to ensure data were available in easily digestible means for use. When the BID Initiative work began in 2014, few systems met the stated system requirements of the countries, and therefore, significant investments were made in the platforms and the development process. Today, the options of these platforms and others would allow for a less intensive investment, both in time and resources.

During the first wave, the primary observations were that the health workers mainly focused on mastering the data collection process with the new tools, but that over time, the data collection process became routine.

During the first wave, health workers mainly focused on mastering the data collection process.

The new tools facilitated improved data collection in the following ways:

- The health workers needed less time to record data. Rather than the duplicative data-entry process required by the legacy paper forms (where health workers had to enter the same data on multiple forms, such as an immunization registry, tally sheets, and stock ledgers), health workers recorded data once and used that data many times, whether to make decisions immediately or to prepare various reports. We conducted a time and motion study in Arusha Region, Tanzania, in 30 health facilities to observe and record the amount of time each health worker spent on activities associated with vaccination, starting from registering the child until administering the vaccine. The study data estimated that health workers spent 41% less time registering and vaccinating each child after introducing the new tools.^16^
- Health workers also saved time preparing monthly reports because the system automatically generated reports and dashboards, saving 2 or more days' worth of work per month from not having to complete monthly report forms. For facilities in Tanzania with automated reporting, health workers saved more than 70 hours or 8 full working days each year that they could allocate to patient care.
- The legacy systems had reinforced a static clinic environment, where health workers had to remain at a table to enter data into large registries and paper forms. The tablets allowed a dynamic and more efficient clinic workflow where the health workers could move with the patient while still entering data.

In general, across users, data completeness and timeliness began to stabilize after the first 3 months of implementation with more consistent level of entries into the systems and a small number of users facing ongoing challenges. Barriers that impeded data collection with the new tools included challenges faced by health workers who had never used a smartphone. Common issues were forgetting passwords or not turning the tablet off after service, which drained the battery. Solar chargers were provided to address challenges with electricity. In addition, maintaining use while health workers were required to continue using the legacy system was critical and challenging.

### Second Wave: Improving Data Quality

During the second wave, health workers strengthened their understanding of data and the importance of data quality being collected, EIR functionality to check data validity, and the requirements to fill in specific data elements. Traditionally, health workers often viewed data collection as a duty for reporting to their superiors and not as something useful to their daily work. As health workers adopted and mastered the new digital systems, we saw them gain a greater understanding of the importance of the quality of the data because they easily saw where there were gaps and errors and the value of more accurate reporting.

As health workers adopted and mastered the new digital systems, we saw them gain a greater understanding of the importance of the quality of the data.

In both countries, PATH used an “on-the-job” (OJT) training methodology^17^ in the health facilities with some mix of training of trainers to incorporate the district-level staff and to build their ability to provide support to the facilities over the long-term. The OJT methodology was built around a series of “touches” or facility visits (from 3–5 visits). The touches began with the introduction to the EIR and the new way of collecting data and progressed to focus on the data use interventions in the later touches as the health workers became comfortable with the tool and could begin to focus on the data now available to them. This training methodology design was based upon John Kotter's Change Model^18^ to anticipate the change process the health workers would experience with the introduction of the new interventions and how they would be motivated or incentivized to use the system and the data produced. OJT training was chosen specifically to build their capacity to use the interventions in their clinical setting and to incorporate them directly into their existing workflows (or adjust workflows as appropriate), as well as to build their motivation by helping show the positive impact on the reduction in reporting time as well as having the data available to support them in their work. The primary observations during the second wave were that as health workers became more familiar with the digital tools (after 2–3 weeks), the data quality improved because the data validation components were integrated into the EIR. For example, before implementation, the availability of tally sheets in the Arusha and Tanga regions of Tanzania was only 52% and 66%, respectively, but after the introduction of the EIR, facilities automatically saw their aggregated counts of vaccinations.^19^ The value placed upon the data increased with the possibility of data access and visibility; as data became more meaningful to the health workers, they were able to focus on how it contributed to their daily work rather than viewing data entry as an isolated activity that they did in addition to delivering immunization services.

The new tools facilitated improvement of data quality in the following ways:

- **Accuracy.** Using the EIR reduced errors that could have occurred when multiple data sources differed without a way to verify the most accurate data point. Accuracy was improved by building data verification into the system itself.
- **Completeness.** The EIR ensured that all critical aspects of the data had to be entered before the health worker could move to the next data point, minimizing information gaps.
- **Efficiency.** With a single point of data entry, the EIR ensured a more efficient workflow and less complicated data collection (such as with multiple paper registries).
- **Timeliness.** Regularly synchronizing the system to a national database improved timeliness. This provided visibility across the health system, as appropriate, and eliminated delays in reporting cycles.

**FIGURE 3:**
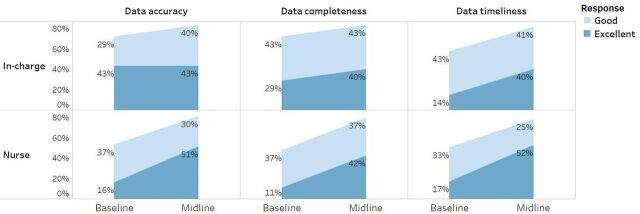
Perceptions of Data Quality Among Facility In-Charges and Immunization Nurses in Tanga Region, Tanzania

In general, across users, known data quality issues reduced and eventually stabilized in the 3–6 months range after implementation.^19^ Initially, the primary barrier to improving data quality was that health workers had to use parallel systems as the new tools were introduced. This double data entry was a high burden on the health workers, affecting quality and completeness in both the legacy system and the new system, making it difficult for health workers to maintain motivation to use both systems as the country government determined their process to transition systems. In particular, according to baseline and midline survey results of health workers in 84 facilities in the Tanga region, perception of quality increased over time as well (Figure 3), potentially due to their increased ability to interact with the data and influence the data quality. Perception of data quality (accuracy, completeness, and timeliness) directly affected the health workers' motivation to use the data as seen in our baseline data where 56% of surveyed facility staff in Zambia cited “poor accuracy” of data as a key barrier to data use.^20^

### Third Wave: Increasing Use of Data for Programmatic Decision Making

The third wave occurred after the new data collection tools had been used for at least 1 month and the health workers had become comfortable with them. At this point, the health workers shifted from solely collecting and sharing data with the district to also demonstrating their use of the data. Key decision areas that were reviewed and discussed with health workers included the ability to calculate coverage in their catchment area, monitor and manage stock levels, and track and trace defaulters. The legacy paper systems had impeded data accessibility and created barriers for significant use of the data in their daily tasks, as cited through health worker interviews at baseline.^19^^,^^20^ If health workers used the data, it had primarily been reactive, focusing on using the data to address issues that arose, such as identifying children who did not come for their vaccinations, ordering stock after it ran low, or having a basic understanding of overall performance.

**Figure:**
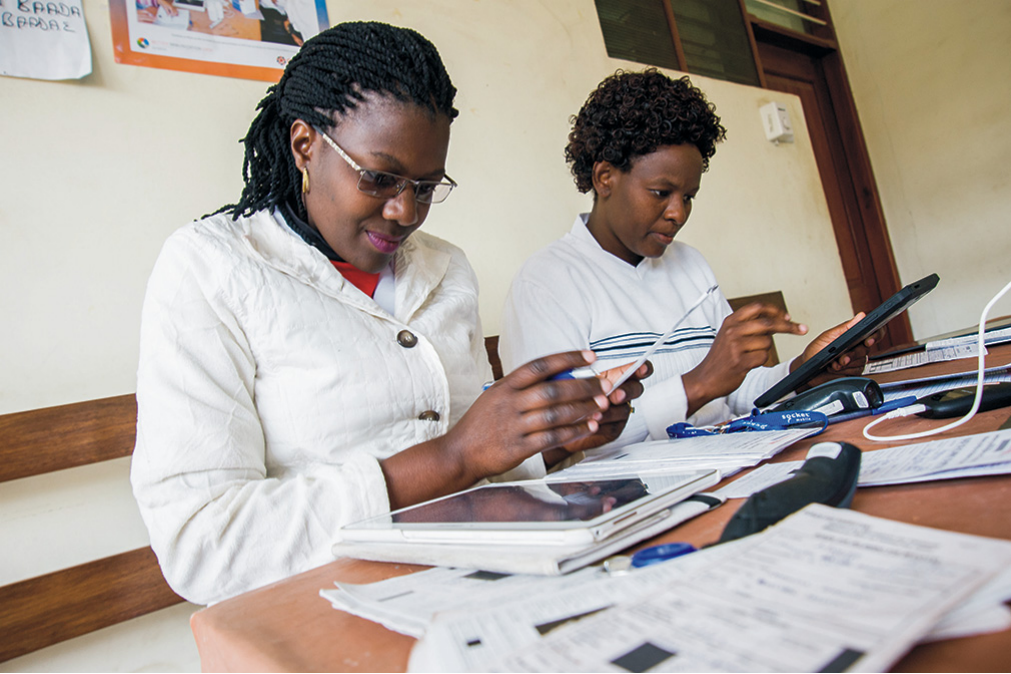
Nurses in Arusha, Tanzania scan child health cards. © 2015 Riccardo Gangale/Bill & Melinda Gates Foundation

During the third wave, health workers shifted from solely collecting and sharing data with the district to also demonstrating their use of the data.

The data use interventions were designed with a user advisory group in each country comprised of health workers from across the health system, with the objective of building the capacity of the health workers to see value in the data and use it to take action to strengthen their work.^21^ These interventions included simple reports within the EIRs interface, such as the “to-do” list, which identifies the children who have missed a vaccine dose for easy follow-up by the health worker. Data use guides helped the facility and district staff by outlining easy-to-assess scenarios using their data to determine actions to take. Supervision coaching tools supported the district staff in using data to address challenges being faced in low-performing facilities. During the third wave, the primary observations were that, as the EIR provided data at their fingertips, health workers could now rapidly and accurately identify the right child and the right dose. Health workers started to use the data to make more proactive routine decisions around service delivery. They transitioned beyond focusing on data collection to using the reports and data visualizations to plan their daily activities, and they began to see trends that could inform ways to strengthen their work.

The EIR and other interventions facilitated data use for decision making in the following ways:

- Empowered health workers at all levels. The EIR encouraged a leadership model where facility in-charges encouraged health workers to make locally appropriate decisions, such as identifying and following up with defaulters or flagging orders for additional stock.
- Enabled ongoing supportive supervision by ensuring that district staff had access to the data needed to evaluate facilities' performance against district expectations, as well as equipping them with appropriate questions to scrutinize the data and address performance gaps.
- Supported peer networking using digital applications so that health workers shared challenges and experiences and received feedback from peers.
- Established a common understanding across each level of the health system through provision of simple electronic data use guides, targeted at both data producers and users.

**FIGURE 4:**
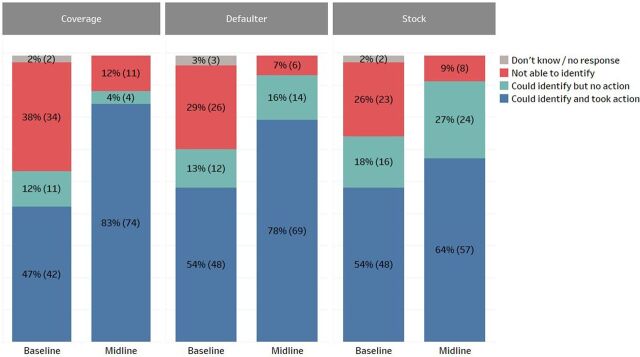
Ability to Identify Low DPT3 Coverage Areas, Defaulters, and Vaccine Stock Levels Among Facility Health Care Workers, Baseline Versus Midline, Southern Province, Zambia Abbreviations: DPT3, third dose of diphtheria, pertussis, and tetanus vaccine.

Users reported increased data use for specific actions, such as identifying defaulters, stock levels, and low DPT3 coverage areas. In Zambia, health workers in Southern Province reported increases in their data use to take action in these 3 areas (Figure 4): a 36% increase in identifying and taking action to address low coverage areas (from 47% to 83%), a 24% increase in taking action on defaulters (from 54% to 78%), and a 10% increase in managing stock levels (from 54% to 64%). These are self-reported results from a survey of health workers at 89 facilities in the first 6 districts of implementation in Zambia; a baseline survey was administered immediately before the implementation of BID interventions and a midline survey was administered approximately 4 months after implementation. The full details of the survey results are reported elsewhere.^19^^,^^20^

Barriers to improving data use for decision making included lack of knowledge transfer during staff rotation and turnover, lack of empowerment for some health workers to make decisions based on the data, and lack of appropriate resources available and the authority to use those resources. If there are no resources to support evidence-based decision making, service delivery can be compromised.

## LESSONS LEARNED

Observing and learning how health workers adopted the new tools and worked through the waves of data use and taking appropriate steps in real time helped ensure successful implementation and results. The following were some key learnings:

- In the early stages, implementers should tailor the trainings to health workers' level of proficiency or lack thereof with digital tools. This aligns with findings from other studies.^13^
- A common misconception was that a new digital tool alone would “solve all problems.”
- Data quality and careful use must be emphasized throughout the process, monitored through supportive supervision, and celebrated when done correctly.^9^
- Consistent use of the tools should be reinforced to have high-quality data accessible for action, such as identifying children who have missed vaccines, reviewing available stock, and summarizing services provided during a specific time frame.
- Implementers should work with the district to identify low-performing facilities and determine which questions to ask about the facilities' data to address performance gaps, encourage feedback loops, and build a more permanent culture of data use.^11^
- All health workers and district staff should be trained in the tools to allow the presence of multiple staff to support any new staff to use the tools consistently. Having all staff trained also mitigates loss of knowledge when health workers leave or transfer among facilities.
- Resistance to change among health workers can affect facility-wide adoption. Understanding how health workers effectively created or embraced change previously in their work can be useful in helping them adopt to new processes and tools. A clear change-management plan to usher health workers through the new systems is also important.
- Supportive supervision (from implementers and supervisors) is needed to follow-up on areas of poor-quality data and discrepancies in the data. Sustainability of the third wave,—institutionalized data use for decision making—requires an empowering leadership model and adequate resources to carry out decisions and actions determined from data use.
- Having a digitally proficient workforce, a policy that allows legacy systems to be retired quickly, strong supportive supervision throughout, and motivated individuals or communities allows the health workers to progress through these waves more quickly.

## CONCLUSION

When tools for health service delivery are redesigned or new, it is important to consider how the tools can impact data quality and use and how the impact unfolds over time or, as seen with BID, progresses across multiple waves. Through the observations conducted under the BID Initiative work, we saw that familiarity with and consistent use of a tool took time, especially when a legacy system was being used in parallel (first wave). However, as more data and complete data sets were entered into the tool, health workers began to appreciate the availability and completeness of the data (second wave). Finally, health workers began to focus on using the data to identify gaps and make decisions in reaction to those gaps, and the district used the data to make decisions around the support they provided to facilities and the allocation of stock (third wave).

As Tanzania and Zambia continue to use these tools to strengthen a culture of data use, we anticipate movement to a fourth wave. In a fourth wave, health workers and immunization managers can shift from a reactive to a proactive approach, using the data to anticipate and plan for challenges before they occur. For example, rather than planning stock for the following month based on the number of children vaccinated in the prior month (third wave), in a fourth wave, we would see health workers noticing trends such as surges of children during certain seasons or times of the year. The health workers would then order vaccines and resources ahead of those seasons or times to ensure the facility could manage and vaccinate the increased number of patients they would expect to see.

In a fourth wave, health workers can shift from a reactive to a proactive approach, using the data to anticipate and plan for challenges before they occur.

In future research, we recommend exploring the sustainability of the first 3 waves and testing the following questions on data use in this fourth wave:

- How do limitations in time, capacity, and sufficient data affect the ability to progress to the fourth wave?
- Do health workers use the data dashboards to look beyond 1 month of service delivery and identify longer-term trends or plan for the impact of large events, such as elections?
- How will facilities maintain knowledge despite staff turnover?

As anticipated and observed by the BID Initiative, a natural progression exists in relation to strengthening data quality and use. Improved data collection tools and building data use capacity are vital for this evolution. Allowing time for health workers to proceed through the waves will ideally allow for creation of a stronger culture of data use—a foundation to be established, maintained, and built upon over time.

## Notes

**Peer Reviewed**

**Funding:** This work was funded through a grant provided by the Bill & Melinda Gates Foundation.
